# Preliminary data on oxytocin modulation of neural reactivity in women to emotional stimuli of children depending on childhood emotional neglect

**DOI:** 10.1002/dev.22349

**Published:** 2022-11-25

**Authors:** Isabell M. Meier, Estrella R. Montoya, Hannah Spencer, Sofia C. Orellana, Mariët van Buuren, Jack van Honk, Peter A. Bos

**Affiliations:** ^1^ Department of Experimental Psychology Utrecht University Utrecht The Netherlands; ^2^ Research Institute of Child Development and Education University of Amsterdam Amsterdam The Netherlands; ^3^ Department of Psychiatry University of Cambridge, Cambridge Biomedical Campus Cambridge UK; ^4^ Department of Clinical, Neuro and Developmental Psychology, Institute for Brain and Behavior Amsterdam Vrije Universiteit Amsterdam Amsterdam The Netherlands; ^5^ Department of Psychiatry and Mental Health, Groote Schuur Hospital, MRC Unit on Anxiety & Stress Disorders University of Cape Town Cape Town South Africa; ^6^ Institute of Infectious Diseases and Molecular Medicine University of Cape Town Cape Town South Africa; ^7^ Institute of Education and Child Studies Leiden University Leiden The Netherlands

**Keywords:** caregiving, childhood emotional neglect, fMRI, oxytocin, salience

## Abstract

Sensitivity for rewarding cues and distress signals from children is fundamental to human caregiving and modulated by the neuropeptide oxytocin. In a functional magnetic resonance imaging study, we investigated whether oxytocin regulates neural responses to reward or distress cues form children. In a placebo‐controlled, within‐subject design, we measured neural responses to positive, negative, and neutral cues from children in 22 healthy female subjects who received oxytocin (24 IU) versus placebo. Further, based on current literature, we hypothesized that oxytocin effects are modulated by experiences of childhood trauma. The task elicited valence‐specific effects—positive images activated the ventromedial prefrontal cortex, left anterior cingulate cortex, and right putamen, and images of children in distress activated the bilateral amygdala, hippocampus, and right medial superior frontal cortex. The effects of oxytocin depended on subjective reports of childhood emotional neglect. Self‐reported neglect interacted with oxytocin administration in the amygdala, hippocampus, and prefrontal areas. In individuals with higher scores of emotional neglect, oxytocin increased neural reactivity of limbic structures to positive and neutral images. Our findings need replication in larger samples and can therefore be considered preliminary but are in line with the recent literature on the modulating effect of childhood adversity on the sensitivity to oxytocin administration.

## 1 INTRODUCTION

Two important components of human parental caregiving are sensitivity for rewarding signals from children, which is highly relevant for communication and bonding, and empathic responses to needs of children. A key modulator of sensitivity for social reward and empathy is the neuromodulatory peptide oxytocin (OXT) (Bethlehem et al., 2014; Bos et al., 2012). OXT has previously been shown to modulate neural sensitivity to rewarding cues from infants, for example, infant laughter and pictures of happy infants (Bos et al., 2018; Gregory et al., 2015; Riem et al., 2012) and neural markers of empathy in response to cues of infant distress such as infant crying (Riem et al., 2011).

Empathy refers to the capacity to automatically read, understand, and react to feelings and intentions of others based on their bodily signals. As such, empathy is an ability crucial for communication as well as formation and maintenance of the parent–infant bond (Bos, 2017; Decety, 2011b; Feldman, 2017). OXT seems to affect certain aspects of empathy, such as the recognition of emotions (Bartz et al., 2010; Leppanen et al., 2017) and the recognition of internal states of others (Domes et al., 2010; Hurlemann et al., 2010), and has further shown to increase empathy for pain on a subjective level, although only in specific contexts (Abu‐Akel et al., 2015; Shamay‐Tsoory et al., 2013). Seemingly in contrast, neural activation in response to physical pain in others was found to be reduced after administration of OXT (Bos et al., 2015), which could be explained by a reduction of neural markers related to personal distress in response to the high aversiveness of the stimuli used in the study.

Furthermore, the effects of OXT do not only vary depending on task selection but are also strongly determined by context and individual characteristics (Bartz et al., 2011; Olff et al., 2013; Shamay‐Tsoory & Abu‐Akel, 2016). Exposure to childhood adversity is one such characteristic that can affect OXT function. Adverse childhood events can comprise neglect and abuse on an emotional and physical level, as well as chronic exposure to stress. Especially, adverse events during early childhood can result in long‐lasting changes in neural, endocrinological, and social functioning (Bos, 2017; Lanius et al., 2010), hence increasing vulnerability to stress and disease (Heim et al., 2008) and negatively affecting parental behavior (Bos, 2017). Part of these effects could be mediated by alternations in functioning of the OXT system highlighting the importance to understand the interaction between these factors. Indeed, early life adversities were found to be associated with decreased OXT cerebrospinal fluid concentrations in women and nonhuman primates (Heim et al., 2009; Strathearn, 2011) and with decreased OXT plasma levels in men (Opacka‐Juffry & Mohiyeddini, 2012). Children who experienced severe emotional neglect early on showed no change in OXT levels after physical contact with their mother, whereas children who were raised in a caring environment showed an increase of OXT (Fries et al., 2005). Neural responses to OXT administration also seem to be dependent on early life adversities, which has repeatedly been shown in male participants in studies relating to stress reactivity (Fan et al., 2015; Grimm et al., 2014; Meinlschmidt & Heim, 2007). In a stress‐related task, subjects with history of early life adversities showed enhanced task‐related effects in limbic structures after OXT administration, whereas controls showed a reduction (Grimm et al., 2014). Another study in the same sample showed that the degree of early life adversities was positively related to amygdala–hippocampal functional connectivity during the stress task, which then was attenuated by oxytocin (Fan et al., 2015). A study investigating resting‐state connectivity in women found OXT to induce changes in functional connectivity in the default mode network, which were absent in individuals who experienced rejecting caregiving experiences (Riem et al., 2013). Besides these studies, the significance of early life adversities on OXT effects in neural responses related to caregiving motivation has not been studied extensively.

In addition to early childhood adversities, OXT effects have been shown to be sex dependent, for example, amygdala reactivity to fearful faces after OXT was found to be enhanced in women, but decreased in men (Domes et al., 2010; Lieberz et al., 2020). Therefore, in the current study we critically address the effects of OXT on the empathic neural response to children in a functional neuroimaging setup in women only. The study was part of a larger project investigating the hormonal precursors of parental caregiving (Bos et al., 2018). We developed a new neuroimaging paradigm to investigate the effect of OXT on sensitivity for social reward and affective empathic responses toward children. The stimuli depicted children in situations of emotional distress and positive social interactions as well as children in neutral situations serving as a control condition. Based on independent subjective evaluations, stimuli of children in emotional distress were selected to elicit sympathetic but not aversive responses (Bos et al., 2021). We will address the effects of OXT versus placebo on neural and subjective responses to the differently valanced stimuli, and further assess whether the effects of OXT depend on interindividual differences in history of childhood emotional neglect and caregiving motivation. OXT (24 IU) was administered intranasally in a placebo‐controlled, randomized within‐subject design. In an additional task postscanning, participants were asked to give a subjective empathy rating of the images they had seen in the scanner.

With OXT being crucial for caregiving responses, it is central to understand the effects of early stress on the OXT system and functional consequences on underlying mechanisms of caregiving responses. Based on the social salience theory of oxytocin (Bartz et al., 2011; Bethlehem et al., 2014; Shamay‐Tsoory & Abu‐Akel, 2016), it can be hypothesized that OXT will increase the neural response to images with children in positive social interactions in structures related to reward, salience, and cognitive empathy, including the nucleus accumbens (Nacc), ventromedial prefrontal cortex (vmPFC), ventral tegmental area (VTA), putamen, caudate nucleus, and thalamus. However, recent work from our own lab in this same sample of women showed reduced activation in neural responses toward infant faces after OXT in areas related to reward and salience. This effect was modulated by caregiving motivation (Bos et al., 2018). It can therefore also be expected that similar responses will be observed toward the positively valenced stimuli. For images depicting children in emotional distress, in line with previous literature of the effects of OXT on emotional reactivity in women (Lieberz et al., 2020), we expect OXT to increase neural reactivity in structures involved in negative emotion processing, including the amygdala, hippocampus, anterior insula (AI), anterior cingulate cortex (ACC), and orbitofrontal cortex (OFC). Further, we expect that these OXT effects depend on interindividual differences in history of childhood emotional neglect and caregiving motivation, considering OXT's crucial role in caregiving behavior and our recent findings of OXT effects toward infant stimuli being modulated by caregiving motivation (Bos et al., 2018).

## 2 MATERIAL AND METHODS

### 2.1 Participants

A group of 26 healthy, female participants were recruited at Utrecht University campus to take part in the study. To avoid hormonal fluctuations during the cycle, only women using combined oral contraceptives (OCs; i.e., containing ethinylestradiol and levonorgestrel; Microgynon 30) were included in the study, and no scans were performed during the hormone‐free interval of the OC regimen (Montoya & Bos, 2017). Participants had no history of psychiatric, neurological, or endocrine abnormalities. Participants did not smoke and used no medication other than OCs. Participants were informed not to consume alcohol or other drugs 24 h, and not to eat or drink 1 h, prior to testing sessions. With regard to drug administration (nasal spray), testing sessions were rescheduled when participants experienced a blocked or running nose.

The experimental protocol was approved by the ethics committee of the University Medical Centre Utrecht, in accordance with the latest declaration of Helsinki. All participants gave written informed consent prior to participation in the study and received payment afterward. Due to technical issues during functional magnetic resonance imaging (fMRI) acquisition (artifacts in functional scans, *n* = 4), the final sample consisted of 22 participants (mean age = 20.25; *SD* = 1.33; range = 18–24).

### 2.2 Drug administration

The setup of the study followed a within‐subjects, double‐blind, placebo‐controlled, counterbalanced crossover design in which 24 IU of OXT was administered (Syntocinon nasal spray; Defiante Farmacêutica, S.A.). Participants self‐administered three puffs (4 IU per puff) per nostril under supervision of the experiment leader. The placebo consisted of an NaCl solution produced by the pharmacist of the University Medical Centre Utrecht in accordance with GCP guidelines.

### 2.3 Procedure

Participants were scanned on two separate days, always at the same time of day in a time frame between 12:00 p.m. and 5:00 p.m., with a minimum interval of 72 h between sessions. Before drug administration, participants were screened for alcohol and drug use, were given brief explanation of the task, and gave written informed consent.

Participants self‐administered the nasal spray under supervision of the experiment leader and were seated in a waiting room until asked to proceed to the scanner. Before entering the scanner, participants were screened using an MRI checklist and a metal detector, and subsequently they were instructed to position themselves on the scanner bed as comfortable as possible and to relax. Foam pads placed between the radiofrequency (RF) coil and the participant's head were used to minimize head movement. Instructions and task images were displayed on an MRI‐compatible monitor placed at the headend of the scanner visible via an angled mirror attached to the coil. Further communication between participant and experiment leader during the scan session was done via intercom. Average time interval between OXT administration and the start of the task was 59 min (*SD* = 5.47), which is in line with most studies showing effects of OXT on behavior so far (Bos et al., 2012). At the end of the second session, participants were asked to guess on which day they received OXT. They were not aware which day they received OXT or placebo (binomial: *p* > .05). Last, all participants were debriefed and received payment.

To control for mood effects of OXT, the Positive and Negative Affect Schedule (PANAS; Watson et al., 1988) was filled out twice, once before administration upon arrival in the laboratory and once directly before proceeding to the scanner (∼30 min after administration).

After the second day of testing, participants were emailed with the request to fill out a Dutch online version of the Childhood Trauma Questionnaire—Short Form (CTQ) (Thombs et al., 2009) and Parental Care and Tenderness (PCAT) questionnaire (Buckels et al., 2015). The CTQ measures childhood maltreatment on five separate constructs, namely, emotional and physical neglect, and emotional, physical, and sexual abuse (Bernstein et al., 2003). The subscale that we considered for the current study, emotional neglect (CTQ‐EN), is based on the definition of “the failure of caretakers to meet children's basic emotional and psychological needs, including love, belonging, nurturance, and support” (Bernstein et al., 2003). We chose this subscale of the CTQ based on the findings of a previous administration study in relation to infant stimuli (Bos et al., 2014). Mean score of our sample on the CTQ‐EN was 8.1 (*SD* = 2.65; range = 5–15), which is comparable with scores obtained in the nonclinical sample of the Dutch validation study (Thombs et al., 2009). Cronbach's *α* for the CTQ‐EN in the Dutch validation study was 0.91 including clinical and nonclinical samples (Thombs et al., 2009).

The PCAT measures parental care motivation that consists of the conceptually separate constructs nurturance and protection, where the former uniquely predicts sensitivity for infant cuteness, and the latter predicts restrictive parenting practices and harsh moral judgments on moral transgressions (Buckels et al., 2015; Hofer et al., 2018). This questionnaire was initially included with relation to an infant cuteness paradigm also part of the fMRI session (see Bos et al., 2018). Since nurturance scores moderated effects of OXT in that study, we ran exploratory analyses for the effects of nurturance tendencies on the current data, reported in the Supporting Information. Similarly, a recent study that used the same neutral and negative stimuli as in the current study showed a modulation of protection scores on behavioral effects of testosterone administration in response to the stimuli. An exploration of effects of protection tendencies has therefore also been added to the Supporting Information.

### 2.4 Empathy task and behavioral ratings

The empathy task, a passive viewing task, was designed to measure empathic responses to positive and negative scenarios. Participants were presented with different luminance‐controlled black‐and‐white stimuli depicting children in scenes with an emotionally negative, positive, or neutral context. Positive stimuli showed rewarding social interactions, children playing together, or parent–infant interactions. Negative images showed children in distressing scenes, for example, an isolated crying child. Based on independent subjective evaluations, stimuli were selected that elicit sympathetic, but not aversive, responses to limit the possibility of personal distress in our participants, which might occur observing physical pain in others. Neutral images depicted children executing a task or playing with toys, with a neutral facial expression and without social interaction. Stimuli were preselected through a rating procedure from a larger set of 120 images obtained after an internet search in a pilot study. Images were rated on empathic content and positivity by 24 female participants (mean age = 34.45, *SD* = 15.03) and in total 24 stimuli were selected per condition based on consistency of the ratings. The resulting conditions vary significantly on empathic content (*F*(2,46) = 452.51, *p* < .001, *η* ^2^ = .95) and positivity (*F*(2, 46) = 326.59, *p* < .001, *η* ^2^ = .93).

A total of 72 images, 24 images per condition, were presented to the participant in a block design, with each image being presented twice but randomized and never within the same block. This resulted in a total of 18 blocks, divided into six blocks per condition. Every block lasted 24,000 ms and consisted of eight stimuli of the same valence, each presented for 3000 ms. A fixation cross was displayed at the start of the task and at the beginning of each block for the duration of 5500 ms.

Participants were instructed to attend the images presented to them carefully. The same task using the same stimuli was performed in both sessions. At the end of each fMRI session, outside of the scanner, participants performed an additional rating task containing a random selection of 36 pictures from the same stimuli, 12 of each condition, on how much compassion (Dutch: “medelijden”) they felt for the child in the image on a 10‐point Likert scale (0 = *No feeling of compassion*; 9 = *Very strong feeling of compassion*). Participants never rated the same stimulus twice during the two sessions. The rating scores were used to perform an exploratory behavioral analysis.

### 2.5 Scanning parameters

Scanning was performed on a 3‐Tesla Philips Achieva MRI scanner (Philips Medical Systems, Best, The Netherlands). Before the functional scans, a high‐resolution anatomical T1‐weighted scan with the following parameters was obtained for co‐registration and normalization purposes: 3.8 ms echo time, 8.4 ms repetition time, 288 × 288 × 175 mm field of view, 175 sagittal slices, flip angle of 8.0°, voxel size of 1.0 mm isotropic.

Blood oxygen level‐dependent (BOLD) response was measured with functional T2*‐weighted axial whole‐brain images. In each session, 490 volumes were acquired during the task using a 2D‐EPI‐SENSE sequence with 51 slices, a flip angle of 65°, voxel size of 2.5 mm isotropic, a SENSE factor of *R* = 3.0 (anterior–posterior), and a field of view of 220 × 127.5 × 220 mm. Repetition time (TR) for volume acquisition was set to 1.01 s and the echo time (TE) to 24 ms.

### 2.6 Preprocessing and data analyses

Preprocessing and subsequent analyses were performed with SPM12 (http://www.fil.ion.ucl.ac.uk/spm). Functional scans of both sessions were realigned after which the anatomical scan was coregistered to the mean functional scan. Subsequently, using unified segmentation, the structural scan was segmented and normalization parameters were estimated. Using these normalization parameters, all volumes were normalized to a standard brain template (MNI) and were resliced at 2 mm isotropic voxel size. Smoothing with a 6‐mm full width at half maximum Gaussian kernel was applied to the normalized functional volumes. Next, within both sessions, a general linear model was applied to the data to investigate the effects of stimulus conditions. Neural responses to the different conditions of emotional valence were modeled using a 24s boxcar function convolved with a hemodynamic response function (hrf) as implemented in the SPM12 software. Additional regressors of no interest that are entered into the analyses to reduce unexplained variance in the data include realignment parameters and a discrete cosine transform high‐pass filter with a cutoff of 128 s.

Next, contrast maps for the different emotional valence conditions versus baseline of both sessions were entered into a second‐level factorial ANOVA, with drug (OXT vs. placebo) and emotional valence (negative, neutral, and positive) as within‐subject factors. *F*‐tests were run to test for the effect of condition (emotional valence), drug, and the interaction effect of OXT with condition. To examine the (de)activations of all stimuli versus rest, comparative *t*‐tests were performed. Further, to test for emotion specific effects, comparative *t*‐tests were performed for the contrasts negative > neutral and positive > neutral, with the data from both sessions (OXT and placebo) combined.

To control for multiple comparisons in the whole‐brain analyses, a threshold was set at *p* < .05 (family‐wise error [FWE] corrected). Additionally, small volume corrections (SVC; *p* < .05 FWE) were applied for the predefined regions of the interest (ROIs): the amygdala, hippocampus, AI, ACC, OFC, putamen, caudate nucleus, and thalamus were based on the automated anatomical labeling (AAL) template (Tzourio‐Mazoyer et al., 2002). The VTA, the Nacc, and the vmPFC are not included in the AAL atlas as masks and were therefore computed based on previous empirical papers and a meta‐analysis. The mask for the VTA was based on Groppe et al. (2013) and consists of two spheres of 10‐mm radius around MNI coordinates *x*, *y*, *z* = ± 9, −18, −18. The bilateral mask for the Nacc was retrieved from Montoya et al. (2014) and based on T1‐weighted scans of 60 separate individuals, normalized in MNI152 space and averaged. The mask for the vmPFC is based on a meta‐analysis on theory of mind by Bzdok et al. (2012) built with a 10‐mm sphere around the MNI coordinates *x*, *y*, *z* = 0, 52, −12. For all predefined anatomical ROIs that showed significant effects in the emotion specific *t*‐contrasts (negative > neutral; positive > neutral) on whole brain level, percentage signal change was extracted using MarsBaR (Brett, 2002) for further exploratory analyses.

### 2.7 Analysis of interindividual differences

To investigate the effect of the CTQ‐EN on the neural responses toward images of different emotional valences as well as the interaction of CTQ‐EN with OXT administration, exploratory analyses were conducted. The extracted values of each ROI were entered into a separate 2 × 3 ANOVA with drug (OXT and placebo) and condition (negative, neutral, and positive) as within‐subject factors and the respective questionnaire as a covariate. Subsequently, CTQ‐EN scores were correlated with the extracted ROI parameters to describe the interaction effects.

### 2.8 Analysis of behavioral data

Behavioral rating data were first screened for outliers, defined as scores higher than 3 *SD* above the mean score, in each condition separately. Based on this criterion, all 22 participants were included in the analysis. Next, scores were averaged over trials by condition (negative, neutral, and positive) and drug (OXT vs. placebo), tested for normality with a Shapiro–Wilk test and then entered in a 2 (drug) × 3 (emotional valence) nonparametric analysis of variance (Friedman test). SPSS 23 (IBM analytics) was used for statistical analysis of the behavioral data and an *α* of 0.05 was applied to test for significance.

## 3 RESULTS

### 3.1 Behavioral data

A 2 (OXT vs. placebo) × 3 (negative, neutral, positive) Friedman's ANOVA revealed a statistically significant difference between empathy ratings, depending on image valence and drug (*χ* ^2^(5) = 93.33, *p* < .0001). Post hoc analyses were conducted using Wilcoxon signed rank tests with Bonferroni correction applied, with the statistical significance level set at *p* < .009. To test for task effects, pairwise comparisons in the data from both sessions (OXT and placebo) combined showed that all conditions differed significantly from each other on ratings of compassion (negative vs. neutral: *Z* = −4.1, *p* < .001; positive vs. neutral: *Z* = −4.1, *p* < .001; negative vs positive: *Z* = −4.1, *p* < .001). Negative images (*M* = 7.56, *SD* = 0.79) were rated significantly higher on compassion than positive (*M* = 0.36, *SD* = 0.38) and neutral ones (*M* = 1.13, *SD* = 0.83), and neutral images higher than positive ones. To test for OXT administration effects on ratings, pairwise comparisons showed a marginally significant effect for ratings of neutral images (*Z* = −2.32, *p* = .02), which did not survive the threshold of .009, with lower ratings after OXT (*M* = 1.003, *SD* = 0.90) compared to placebo (*M* = 1.25, *SD* = 0.88). All other *p*‐values were >.30.

### 3.2 Mood data

To test for possible effects of OXT on mood within and between the two testing sessions, the sum scores on the positive and negative affect scale of the PANAS (Watson et al., 1988) were entered into a 2 × 2 repeated measures ANOVA with drug (OXT, placebo) and time of measurement (before and after drug administration) as within subject factors. OXT did not affect mood ratings on the positive (*F*(1,21) = .001, *p* = .974, *η* ^2^ = .00) and negative (*F*(1,21) = .011, *p* = .918, *η* ^2^ = .001) scale, nor did it interact with the time of mood measurement for positive (*F*(1,21) = .981, *p* = .333, *η* ^2^ = .05) or negative (*F*(1,21) = 3.36, *p* = .081, *η* ^2^ = .14) affect scores. Overall mean scores on the PANAS were *M* = 34.4 (*SD* = 4.9) for the positive affect scale and *M* = 12.8 (*SD* = 2.8) for the negative affect scale.

### 3.3 Functional neuroimaging data

In the full factorial analysis on whole brain level, the *0*‐test for main effect of condition resulted in extensive activation including bilateral visual cortices, fusiform gyrus, inferior parietal and temporal gyri, the precuneus, and the medial superior frontal cortex, including the following of our ROIs: hippocampus, amygdala, insula, ACC, vmPFC, and thalamus (see Table 1; Supporting Information). VTA, Nacc, putamen, and caudate nucleus showed no significant activation on whole‐brain or ROI level.

**TABLE 1:** Overview of the peak *T*‐ and *F*‐values, *p*‐values, cluster sizes, and MNI coordinates for significantly activated voxels

Experimental effect		Peak voxel location					
Region		*X*	*Y*	*Z*	*F*/*T* value	Cluster size	*p*‐values
Full factorial							
F‐test: Main effect of condition							
Temporal mid R	R	48	–68	8	35.34	302	<0.001*
Occipital mid L	L	–46	–76	10	32.42	266	<0.001*
Fusiform gyrus	L	–24	–52	–14	29.94	84	<0.001*
Precuneus L	L	–2	–58	44	28.70	316	<0.001*
Inferior temporal gyrus	L	–52	–56	–12	26.25	64	<0.001*
Fusiform gyrus	R	28	–46	–14	23.27	28	<0.001*
Precuneus R	R	–16	–58	62	23.22	23	<0.001*
Hippocampus	L	–16	–6	–12	22.19	19	<0.001*
Inferior parietal lobe	L	–48	–34	44	21.51	79	<0.001*
Vermis 3		–2	–32	–4	20.96	15	<0.01*
Occipital mid L	L	–10	–92	0	20.42	40	<0.01*
Lingual gyrus	L	–16	–66	–6	20.21	7	<0.01*
Hippocampus	R	18	–6	–10	19.18	3	<0.01*
Inferior frontal gyrus	R	36	32	10	19.02	7	<0.01*
Fusiform gyrus	L	–32	–32	–24	18.38	3	<0.05*
Precentral gyrus	L	–50	4	34	17.76	4	<0.05*
Hippocampus	L	–30	–28	–8	17.36	1	<0.05*
Medial superior frontal gyrus	R	6	50	40	16.86	1	<0.05*
Occipital mid L	L	–32	–70	22	16.70	3	<0.05*
Cuneus R	R	4	–74	26	16.43	2	<0.05*
Amygdala	L	–18	–4	–14	19.11	59	<0.01**
Amygdala	R	22	–6	–12	14.29	12	<0.01**
Parahippocampal gyrus	R	30	–4	–28	9.45	3	<0.05**
Insula	R	36	32	8	15.33	2	<0.01**
Insula	L	–26	14	–16	12.49	1	<0.05**
Anterior cingulate cortex	L	–4	50	10	11.06	1	<0.05**
Ventromedial prefrontal cortex	L	–4	54	–10	14.86	54	<0.001**
Thalamus	L	–2	–14	0	12.79	5	<0.01**
Thalamus	R	0	–12	6	10.40	1	<0.05**
*F*‐test: Interaction drug × condition							
Superior frontal cortex	L	–22	56	6	16.28	1	<0.05*
*T*‐test: Positive > Neutral							
Occipital mid	L	–10	–92	0	6.38	107	<0.001*
Occipital mid	L	–48	–76	12	6.19	47	<0.001*
Occipital mid	R	46	–76	2	6.10	26	<0.001*
Occipital mid	R	40	–68	18	5.78	61	<0.01*
Ventromedial prefrontal cortex	L	–4	54	–10	5.44	3	<0.05*
Precuneus cortex	L	0	–56	42	5.36	32	<0.05*
Precuneus cortex	R	6	–56	52	5.10	1	<0.05*
Anterior cingulate cortex	L	–4	50	10	6.64	16	<0.01**
Anterior insula	L	–26	14	–16	4.60	2	<0.01**
Putamen	R	22	6	10	4.96	14	<0.001**
Putamen	R	28	8	4	3.98	1	<0.05**
Putamen	R	30	6	2	3.91	1	<0.05**
*T*‐test: Negative > Neutral							
Hippocampus	L	–16	–6	–14	6.56	38	<0.001*
Hippocampus	R	18	–6	–10	6.19	15	<0.001*
Vermis 3		0	–32	–2	6.13	17	<0.001*
Hippocampus	L	–30	–28	–8	5.86	11	<0.01*
Hippocampus	R	30	–24	–8	5.54	6	<0.01*
Hippocampus	R	24	–14	–12	5.22	4	<0.05*
Medial superior frontal gyrus	R	6	50	40	5.10	1	<0.05*
Hippocampus	R	32	–28	–10	5.09	1	<0.05*
Amygdala	L	–18	–4	–14	6.16	16	<0.001**
Amygdala	R	22	–6	–12	5.18	1	<0.001**

Abbreviations: L, left; R, right.

^∗^ Whole brain FWE corrected at cluster level

^∗∗^ small volume FWE corrected at cluster level.

**FIGURE 1:**
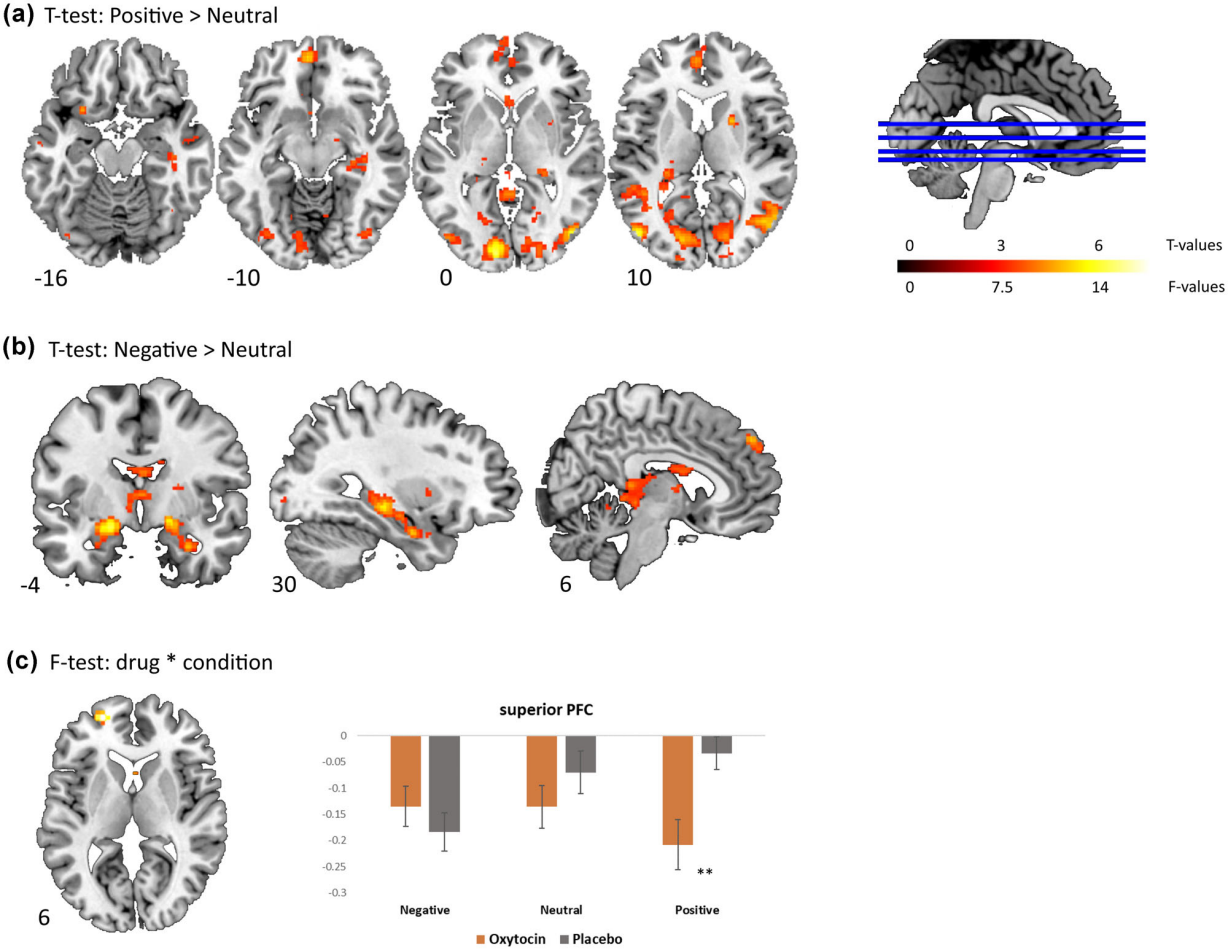
(a) Axial slices with corresponding *Z*‐coordinates (MNI) from the T‐map of neural activation for positive versus neutral images, depicting significant activation in the left anterior insula, left ventromedial prefrontal cortex (vmPFC), left anterior cingulate cortex (ACC), and right putamen, overlaid onto a standard anatomical template. (b) One coronal and two sagittal slices with corresponding *X*‐ and *Y*‐coordinates (MNI), respectively, from the T‐map of neural activation for negative versus neutral images. Significant activation of bilateral amygdala, bilateral hippocampus, left thalamus, and right medial superior frontal cortex is depicted. (c) Axial slice with corresponding *Z*‐coordinate (MNI) from the F‐map of neural activation of the interaction of drug × condition, depicting significant activation in the left superior PFC. A bar graph of the parameter estimates, extracted from a 10‐mm sphere around the functional region of interest (ROI) of the left superior PFC, in all conditions versus rest is displayed. Accompanying statistics are described in the text. All statistical maps are thresholded at *p* = .001 uncorrected, for illustration purposes only. **p = .002

Across both drug conditions, *t*‐tests for the specific effect of positive > neutral (Figure 1a; Table 1) showed significant activation in occipital areas (*p* < .01), the left vmPFC (*p* < .05), and the bilateral precuneus (*p* < .05). Further analyses within our a priori ROIs resulted in significant activation of left ACC (*p* < .01, SVC), left anterior insula (*p* < .01, SVC), and right putamen (*p* < .001, SVC). The *t*‐test for negative > neutral (Figure 1b; Table 1) resulted in significant activation of the bilateral hippocampus (*p* < .05) and the right medial superior frontal cortex (*p* < .05). In addition, we observed significant activation in the bilateral amygdala (*p* < .001, SVC) and the left thalamus (*p* < .001, SVC).

Regarding the drug administration, no overall main effect of OXT was found on whole brain level and in any of the ROIs. We did, however, find a significant interaction of drug × condition in the superior prefrontal cortex (sPFC), although only one voxel exceeded the whole brain FWE correction (−22, 56, 6; *p* < .05, FWE; Figure 1c; Table 1). Based on the significant interaction, a functional ROI of the sPFC was defined. A repeated measures ANOVA on the extracted values of that functional ROI resulted in a significant interaction of drug × condition (*F*(2,42) = 12.24, *p* < .0001, *η* ^2^ = .37), confirming the result found on whole brain level. The results further indicated a decrease of activation after OXT in the positive (OXT: *M* = –0.208, *SD* = 0.048; placebo: *M* = –0.034, *SD* = 0.031) and neutral condition (OXT: *M* = –0.136, *SD* = 0.041; placebo: *M* = –0.07, *SD* = 0.04) and an increase in the negative condition (OXT: *M* = –0.135, *SD* = 0.039; placebo: *M* = –0.183, *SD* = 0.037). Post hoc paired sample *t*‐tests on the extracted values of the sPFC ROI showed a significant decrease of activation after OXT in the positive condition (*t*(21) = −3.46, *p* = .002), but no significant change in the negative or neutral condition (*p* = .39 and *p* = .23, respectively). No interaction effects were found in the a priori ROIs (*p*s > .05).

To investigate the effect of individual differences of having experienced childhood emotional neglect, we added the CTQ‐EN scores as a covariate in separate repeated measures ANOVAs with the extracted values of a priori ROIs that were found to be significant in the emotion specific *t*‐tests (positive > neutral; negative > neutral), and the extracted values from the sPFC. The CTQ‐EN significantly interacted with OXT administration in the amygdala (*F*(1,20) = 6.12, *p* = .022, *η* ^2^ = .24) and with OXT × emotional valence in the hippocampus (*F*(2,40) = 3.67, *p* = .034, *η* ^2^ = .155) and the putamen (*F*(2,40) = 3.86, *p* = .029, *η* ^2^ = .162). In the prefrontal regions, CTQ‐EN significantly interacted with OXT administration in the extracted values of the functional ROI in the sPFC (*F*(1,20) = 64.3, *p* = .051, *η* ^2^ = .18) and with OXT × emotional valence in the vmPFC (*F*(2,40) = 5.32, *p* = .009, *η* ^2^ = .21) and the ACC (*F*(2,40) = 3.43, *p* = .042, *η* ^2^ = .147).

**FIGURE 2:**
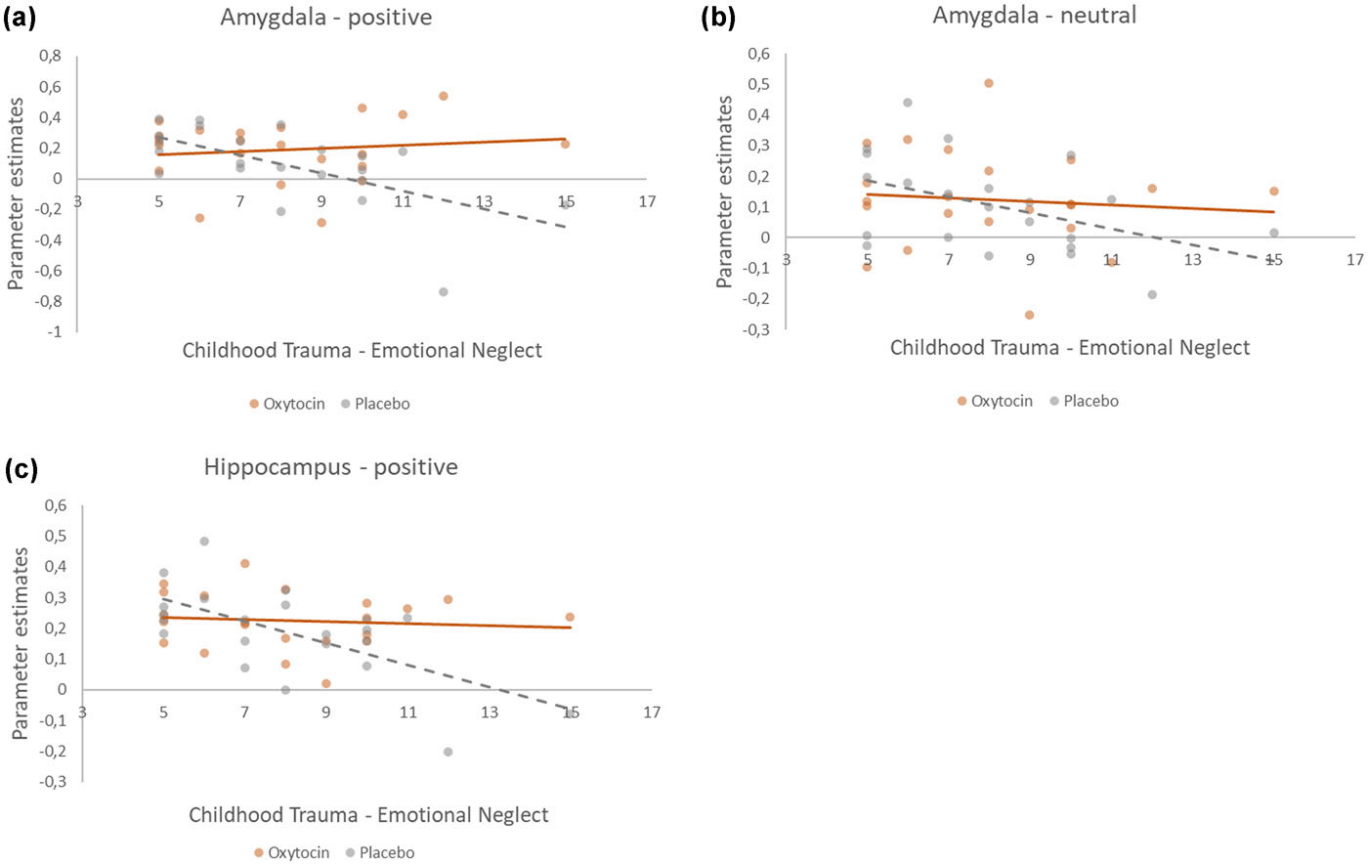
Scatterplots of the correlations between extracted parameter estimates from the anatomical bilateral amygdala toward positive (a) and neutral (b) stimuli and the participant scores of the CTQ‐EN, for oxytocin (OXT) and placebo. Scatterplot of the correlation between extracted parameter estimates from the anatomical bilateral hippocampus toward positive stimuli (c) and the participant scores of the CTQ‐EN, for OXT and placebo. Accompanying statistics are described in the text.

Subsequently, to test for the direction of these effects, the CTQ‐EN was added in a correlational analysis with the extracted values of the a priori ROIs amygdala, hippocampus, vmPFC, ACC, and sPFC. We found significant negative correlations between childhood emotional neglect and activation in the amygdala after placebo in all three conditions (positive conditions: *r* = –.618, *p* = .002; neutral condition: *r* = –.457, *p* = .033; see Figure 2a,b; albeit marginally significant in the negative condition: *r* = –.412, *p* = .056). No such relations were observed in the OXT conditions (positive: *r* = –.091, *p* = .687; neutral: *r* = –.206, *p* = .358; negative: *r* = –.082, *p* = .716). Similarly, a negative correlation between CTQ‐EN and activation in the hippocampus was found for positive images only after placebo (*r* = –.641, *p* = .001), but not after OXT (*r* = –.128, *p* = .570; Figure 2c).

**FIGURE 3:**
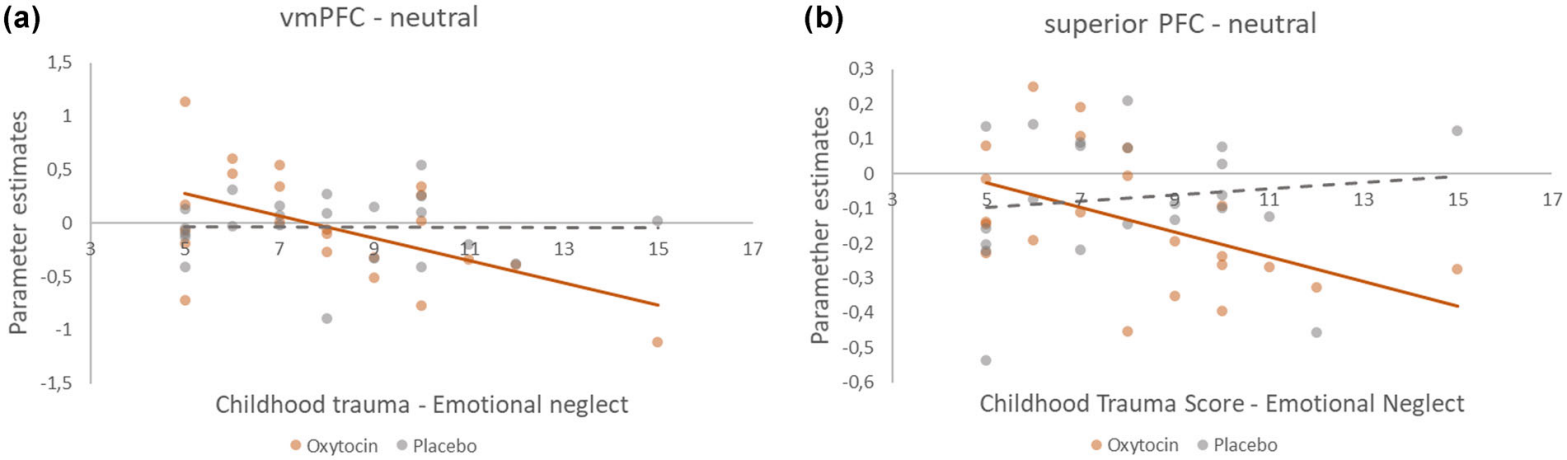
Scatterplot of the correlation between extracted parameter estimates of a 10‐mm sphere around the MNI coordinates 0, 52, −12 (Bzdok et al., 2012) toward neutral stimuli and the participant scores of the CTQ‐EN, for oxytocin (OXT) and placebo (a). Scatterplot of the correlation between extracted parameter estimates from the functional region of interest (ROI) of the superior prefrontal cortex (sPFC) (−22, 56, 6) toward neutral stimuli, and the participant scores of the CTQ‐EN, for OXT and placebo (b). Accompanying statistics are described in the text.

At the same time, negative correlations between childhood emotional neglect and activation in prefrontal ROIs were found in the OXT condition, which did not hold for placebo (Figure 3a,b). We found a negative correlation of CTQ‐EN with activation in the vmPFC after OXT for the neutral condition (*r* = –.539, *p* = .01; not for placebo: *r* = –.01, *p* = .964; Figure 3a) and in the sPFC after OXT for the neutral condition (*r* = –.492, *p* = .02; *r* = .126, *p* = .576; Figure 3b).

No significant correlation was found between CTQ‐EN and ACC, or CTQ‐EN and putamen (all *p* < .05). Hence, emotional childhood neglect was negatively associated with neural activation in amygdala and hippocampus under placebo, whereas this association disappeared under OXT. At the same time, activation in the frontal regions, sPFC and vmPFC, were negatively associated with childhood emotional neglect under OXT exclusively.

### 3.4 Post hoc connectivity analysis

With our results indicating that OXT increases neural activation in amygdala and hippocampus, and simultaneously decreases activation in sPFC and vmPFC in individuals with high emotional neglect scores, we conducted an exploratory connectivity analysis to probe whether OXT administration results in attenuated prefrontal control allowing for an increase of activity in limbic areas (see Supporting Information).

For connectivity from the sPFC seed region to amygdala and hippocampus, no suprathreshold activation clusters were found in the neutral and positive condition in either of the two drug contrasts (OXT > placebo; placebo > OXT). For connectivity between the vmPFC seed region with amygdala and hippocampus, no suprathreshold activation clusters were found for either drug contrast in the neutral condition or the placebo > OXT contrast in the positive condition. In the OXT > placebo contrast, one activation cluster (−22, −32, −6) containing one voxel was found in the hippocampus after SVC. No suprathreshold clusters were detected in the amygdala. In sum, we did not find any significant relationship between connectivity within our ROIs and CTQ‐EN after correction for multiple comparisons.

## 4 DISCUSSION

In this study, we investigated the effects of OXT in a group of healthy nulliparous women on neural responses toward children in positive social situations, or in distressing situations, in a newly developed neuroimaging task. The task evoked emotion‐specific effects on behavior and neural responses. The ratings on how much compassion participants felt with the children validated our task on a subjective level: negative images received significantly higher ratings of compassion than neutral or positive ones. Testing for the effect of OXT administration on subjective feelings of compassion toward the children in the images showed no significant results after correction for multiple comparisons.

On the neural level, images depicting children in positive social situations activated areas related to reward and salience processing (Bos et al., 2015; Seeley et al., 2007) including the vmPFC, precuneus, ACC, anterior insula, and putamen. There was, however, no significant activation of Nacc, caudate, and VTA, core structures involved in the processing of reward (Haber & Knutson, 2010; Peciña et al., 2006). At the same time, images depicting children in distress elicited activation in the hippocampus, amygdala, thalamus, and medial superior prefrontal cortex, areas related to threat and emotion processing as well as affective empathy (Decety, 2011a; Reniers et al., 2014). Overall, the outcome validated our paradigm as an adequate tool to investigate the neural foundation of affective responses to children.

Regarding the effect of drug administration, we did not observe overall effects of OXT in any a priori ROIs or areas that showed task‐related effects, although an interaction effect of OXT and emotional valence was found in the left sPFC. OXT induced a small but significant decrease of activation to positive stimuli in the sPFC. This finding should be interpreted with caution given that the region did not show task‐related effects and did not belong to our a priori ROIs. Previous research found the left sPFC to be mainly involved in working memory load (Boisgueheneuc et al., 2006), and it is thus unclear why it interacted with task condition and OXT administration.

Importantly, we hypothesized that OXT modulation would vary (Bos et al., 2018) with experienced emotional neglect (Fan et al., 2014, 2015; Grimm et al., 2014; Meinlschmidt & Heim, 2007). Childhood experiences of emotional neglect indeed showed differential effects of OXT within the amygdala, hippocampus and prefrontal regions. More specifically, in high‐emotional‐neglect individuals OXT increased neural reactivity to positive and neutral stimuli in the amygdala, and to positive stimuli in the hippocampus. Moreover, in high‐emotional‐neglect participants OXT administration attenuated activation in the vmPFC and sPFC to neutral images, which could suggest a decrease in regulatory prefrontal control to stimuli of neutral content. Existing literature indicates changes of connectivity between amygdala, hippocampus, and prefrontal areas in individuals with early life adversity or posttraumatic stress disorder (PTSD) (Fan et al., 2014, 2015; Frijling et al., 2016). We therefore conducted an exploratory analysis to investigate possible connectivity changes between the vmPFC and sPFC with amygdala and hippocampus in our sample. We did not find any connectivity differences within the hippocampus or amygdala, nor any significant correlation with childhood emotional neglect. The exploratory nature of the analysis and our small sample size do not allow for further interpretation of the connectivity results; however, a replication with a larger sample size could be of value.

While we cannot conclude that OXT impacts prefrontal regulation of amygdala and hippocampus, our results do show, for the first time to our knowledge, that OXT modulates activity in limbic and prefrontal structures to cues of children depending on childhood emotional neglect in women. Our findings are difficult to compare with other studies that look at the interaction of OXT administration and early life adversities. These studies measure stress reactivity or resting state (Fan et al., 2014, 2015) and are mostly conducted in male subjects where sex differences in OXT effects can be expected (Lieberz et al., 2020). It is therefore of interest to consider studies with paradigms more similar to our own study design, which have been conducted in individuals with PTSD or who experienced trauma during adulthood. PTSD and trauma exposure during adulthood are not directly comparable to the relatively modest early life adversities reported in our subclinical sample, nonetheless the following studies are relevant to the interpretation of our findings.

In line with the increase of amygdala reactivity after OXT found in our sample, a study investigating neural reactivity to emotional stimuli in individuals shortly posttrauma found increased amygdala reactivity to negative emotional stimuli after OXT compared to placebo (Frijling et al., 2015). Interestingly, another study found that OXT decreased amygdala reactivity to emotional stimuli independent of valence in a sample of PTSD patients, but enhanced amygdala reactivity to all stimuli in matched healthy trauma‐exposed controls (Koch et al., 2016). The difference in the effect of OXT administration on neural activity between the two populations could be mediated through changes in receptor expression in individuals with PTSD compared to more resilient controls, possibly indicating that trauma severity and resilience to stress might be decisive factors in changes of OXT sensitivity (Nawijn et al., 2019).

Overall, the results suggest that in individuals who experienced emotional neglect, OXT boosts emotion and salience perception of socially rewarding and neutral images of children by increasing activity in amygdala and hippocampus and simultaneously decreasing activity in the vmPFC and sPFC. Being responsive to reward signals from children is highly relevant for communication and bonding, and therefore a crucial aspect of caregiving behavior (Bos, 2017; Feldman, 2017). Although negative cues are already highly salient (as is demonstrated by strong activation of amygdala and hippocampus in the task‐related effects), for high‐emotional‐neglect individuals positive or neutral images might be less salient in comparison. A careful interpretation could therefore be that OXT increases salience of positive and negative stimuli in individuals who are less sensitive to such socially relevant information.

We were surprised not to find effects on the Nacc, caudate, and VTA, since in our previous work on the same sample of women we did show activation in the bilateral VTA as well as putamen, amygdala, insula, and ACC in response to infant faces (Bos et al., 2018). This is most likely due to differences in task stimuli, as the reward function of infant faces is fundamental to human caregiving early in life when infants are most dependent on parental care for survival (Hahn & Perrett, 2014). In addition, previous literature shows variation in activation of the reward system toward social stimuli depending on sex‐, task‐, and stimulus‐related differences (Gregory et al., 2015; Groppe et al., 2013; Riem et al., 2012; Spreckelmeyer et al., 2009). It should equally be taken into account that our sample consisted of OC users. A study investigating the effects of oxytocin on social reward cues found that OXT‐induced reward responses in the VTA and ventral striatum were suppressed in OC users compared to naturally cycling women (Scheele et al., 2016). It is therefore possible that OC use contributed to lacking effects of OXT on VTA and striatum in our study.

We found no effects in areas involved in response to watching physical pain stimuli with a high aversive component (Bos et al., 2015) such as the AI, ACC, or sensorimotor cortices, whereas we do show strong activation of the amygdala, hippocampus, thalamus, and prefrontal areas involved in the processing of negative emotions. Sensitivity of these regions to distress is highly relevant for caregiving behavior as it reflects increased emotion processing and possibly empathic responses (Bos et al., 2014; Feldman, 2017). That we did not find activation in the aforementioned regions involved in empathy for pain might reflect that our stimuli indeed induce feelings of compassion, but are not emotionally aversive, such as the sound of an infant crying, which robustly activates the insula (Witteman et al., 2019). Finally, while our findings are in line with the reported literature (Frijling et al., 2015; Koch et al., 2016), they should be considered preliminary. We suggest a replication of our findings in a larger sample size with more variation in experienced childhood trauma.

In conclusion, the study validated our newly developed neuroimaging task on neural responses to infant cues of different emotional valence. Effects of OXT administration were mostly dependent on the level of self‐reported childhood emotional neglect for our hypothesized regions amygdala, hippocampus, and vmPFC. This study is the first, to our knowledge, to report that exposure to emotional neglect during childhood in a subclinical range in women results in increased neural reactivity of amygdala and hippocampus, and a deactivation of prefrontal areas after OXT. The results suggest that OXT facilitates the processing of rewarding and neutral pictures from infants, which is relevant for how women process affective signals from children, and thus for caregiving behavior. The findings further contribute to literature suggesting a crucial role for OXT in increasing salience perception of social cues, highlighting the importance of individual characteristics.

## CONFLICT OF INTEREST

The authors declare no conflict of interest.

## Supporting information

Supplementary materials

## Data Availability

The data that support the findings of this study are available from the corresponding author upon reasonable request.

## References

[dev22349-bib-0001] Abu‐Akel, A. , Palgi, S. , Klein, E. , Decety, J. , & Shamay‐Tsoory, S. (2015). Oxytocin increases empathy to pain when adopting the other‐ but not the self‐perspective. Social Neuroscience, 10(1), 7–15. 10.1080/17470919.2014.948637 25103924

[dev22349-bib-0002] Bartz, J. A. , Zaki, J. , Bolger, N. , Hollander, E. , Ludwig, N. N. , Kolevzon, A. , & Ochsner, K. N. (2010). Oxytocin selectively improves empathic accuracy. Psychological Science, 21(10), 1426–1428. 10.1177/0956797610383439 20855907 PMC6634294

[dev22349-bib-0003] Bartz, J. A. , Zaki, J. , Bolger, N. , & Ochsner, K. N. (2011). Social effects of oxytocin in humans: Context and person matter. Trends in Cognitive Sciences, 15(7), 301–309. 10.1016/j.tics.2011.05.002 21696997

[dev22349-bib-0004] Bernstein, D. P. , Stein, J. a. , Newcomb, M. D. , Walker, E. , Pogge, D. , Ahluvalia, T. , Stokes, J. , Handelsman, L. , Medrano, M. , Desmond, D. , & Zule, W. (2003). Development and validation of a brief screening version of the Childhood Trauma Questionnaire. Child Abuse and Neglect, 27(2), 169–190. 10.1016/S0145-2134(02)00541-0 12615092

[dev22349-bib-0005] Bethlehem, R. A. I. , Baron‐Cohen, S. , van Honk, J. , Auyeung, B. , & Bos, P. A. (2014). The oxytocin paradox. Frontiers in Behavioral Neuroscience, 8, 1–5. 10.3389/fnbeh.2014.00048 24596548 PMC3925826

[dev22349-bib-0006] Boisgueheneuc, F. D. , Levy, R. , Volle, E. , Seassau, M. , Duffau, H. , Kinkingnehun, S. , Samson, Y. , Zhang, S. , & Dubois, B. (2006). Functions of the left superior frontal gyrus in humans: A lesion study. Brain, 129(12), 3315–3328. 10.1093/brain/awl244 16984899

[dev22349-bib-0007] Bos, P. A. (2017). The endocrinology of human caregiving and its intergenerational transmission. Development and Psychopathology, 29(3), 971–999. 10.1017/S0954579416000973 27760577

[dev22349-bib-0008] Bos, P. A. , Lesemann, F. H. P. , Spencer, H. , Stein, D. J. , van Honk, J. , & Montoya, E. R. (2021). Preliminary data on increased reactivity towards children in distress after testosterone administration in women: A matter of protection? Biological Psychology, 165, 108176. 10.1016/J.BIOPSYCHO.2021.108176 34474128

[dev22349-bib-0009] Bos, P. A. , Montoya, E. R. , Hermans, E. J. , Keysers, C. , & van Honk, J. (2015). Oxytocin reduces neural activity in the pain circuitry when seeing pain in others. NeuroImage, 113, 217–224. 10.1016/j.neuroimage.2015.03.049 25818690 PMC4562366

[dev22349-bib-0010] Bos, P. A. , Montoya, E. R. , Terburg, D. , & van Honk, J. (2014). Cortisol administration increases hippocampal activation to infant crying in males depending on childhood neglect. Human Brain Mapping, 35(10), 5116–5126. 10.1002/hbm.22537 24757127 PMC6868930

[dev22349-bib-0011] Bos, P. A. , Panksepp, J. , Bluthé, R. ‐M. , & van Honk, J. (2012). Acute effects of steroid hormones and neuropeptides on human social‐emotional behavior: A review of single administration studies. Frontiers in Neuroendocrinology, 33(1), 17–35. 10.1016/j.yfrne.2011.01.002 21256859

[dev22349-bib-0012] Bos, P. A. , Spencer, H. , & Montoya, E. R. (2018). Oxytocin reduces neural activation in response to infant faces in nulliparous young women. Social Cognitive and Affective Neuroscience, 13(10), 1099–1109. 10.1093/scan/nsy080 30203082 PMC6204485

[dev22349-bib-0061] Brett, M. , Anton, J. L. , Valabregue, R. , & Poline, J. B. (2002). Region of interest analysis using an SPM toolbox [abstract] Presented at the 8th International Conference on Functional Mapping of the Human Brain, June 2‐6, 2002, Sendai, Japan. Available on CD‐ROM in NeuroImage, Vol 16, No 2.

[dev22349-bib-0060] Buckels, E. E. , Beall, A. T. , Hofer, M. K. , Lin, E. Y. , Zhou, Z. , & Schaller, M. (2015). Individual differences in activation of the parental care motivational system: Assessment, prediction, and implications. Journal of Personality and Social Psychology, 108(3), 497–514. 10.1037/pspp0000023 25559194

[dev22349-bib-0013] Bzdok, D. , Schilbach, L. , Vogeley, K. , Schneider, K. , Laird, A. R. , Langner, R. , & Eickhoff, S. B. (2012). Parsing the neural correlates of moral cognition: ALE meta‐analysis on morality, theory of mind, and empathy. Brain Structure and Function, 217(4), 783–796. 10.1007/s00429-012-0380-y 22270812 PMC3445793

[dev22349-bib-0014] Decety, J. (2011a). Dissecting the neural mechanisms mediating empathy. Emotion Review, 3(1), 92–108. 10.1177/1754073910374662

[dev22349-bib-0015] Decety, J. (2011b). The neuroevolution of empathy. Annals of the New York Academy of Sciences, 1231, 35–45. 10.1111/j.1749-6632.2011.06027.x 21651564

[dev22349-bib-0016] Domes, G. , Lischke, A. , Berger, C. , Grossmann, A. , Hauenstein, K. , Heinrichs, M. , & Herpertz, S. C. (2010). Effects of intranasal oxytocin on emotional face processing in women. Psychoneuroendocrinology, 35(1), 83–93. 10.1016/j.psyneuen.2009.06.016 19632787

[dev22349-bib-0017] Fan, Y. , Herrera‐Melendez, A. L. , Pestke, K. , Feeser, M. , Aust, S. , Otte, C. , Pruessner, J. C. , Böker, H. , Bajbouj, M. , & Grimm, S. (2014). Early life stress modulates amygdala‐prefrontal functional connectivity: Implications for oxytocin effects. Human Brain Mapping, 35(10), 5328–5339. 10.1002/hbm.22553 24862297 PMC6869775

[dev22349-bib-0018] Fan, Y. , Pestke, K. , Feeser, M. , Aust, S. , Pruessner, J. C. , Böker, H. , Bajbouj, M. , & Grimm, S. (2015). Amygdala‐hippocampal connectivity changes during acute psychosocial stress: Joint effect of early life stress and oxytocin. Neuropsychopharmacology, 40(12), 2736–2744. 10.1038/npp.2015.123 25924202 PMC4864649

[dev22349-bib-0019] Feldman, R. (2017). The neurobiology of human attachments. Trends in Cognitive Sciences, 21(2), 80–99. 10.1016/j.tics.2016.11.007 28041836

[dev22349-bib-0020] Fries, A. B. W. , Ziegler, T. E. , Kurian, J. R. , Jacoris, S. , & Pollak, S. D. (2005). Early experience in humans is associated with changes in neuropeptides critical for regulating social behavior. Proceedings of the National Academy of Sciences of the United States of America, 102(47), 17237–17240. 10.1073/pnas.0504767102 16303870 PMC1287978

[dev22349-bib-0021] Frijling, J. L. , van Zuiden, M. , Koch, S. B. J. , Nawijn, L. , Veltman, D. J. , & Olff, M. (2015). Effects of intranasal oxytocin on amygdala reactivity to emotional faces in recently trauma‐exposed individuals. Social Cognitive and Affective Neuroscience, 11(2), 327–336. 10.1093/scan/nsv116 26382634 PMC4733344

[dev22349-bib-0022] Frijling, J. L. , Van Zuiden, M. , Koch, S. B. J. , Nawijn, L. , Veltman, D. J. , & Olff, M. (2016). Intranasal oxytocin affects amygdala functional connectivity after trauma script‐driven imagery in distressed recently trauma‐exposed individuals. Neuropsychopharmacology, 41(5), 1286–1296. 10.1038/npp.2015.278 26346640 PMC4793112

[dev22349-bib-0023] Gregory, R. , Cheng, H. , Rupp, H. A. , Sengelaub, D. R. , & Heiman, J. R. (2015). Oxytocin increases VTA activation to infant and sexual stimuli in nulliparous and postpartum women. Hormones and Behavior, 69, 82–88. 10.1016/j.yhbeh.2014.12.009 25562711 PMC4418634

[dev22349-bib-0024] Grimm, S. , Pestke, K. , Feeser, M. , Aust, S. , Weigand, A. , Wang, J. , Wingenfeld, K. , Pruessner, J. C. , La Marca, R. , Böker, H. , & Bajbouj, M. (2014). Early life stress modulates oxytocin effects on limbic system during acute psychosocial stress. Social Cognitive and Affective Neuroscience, 9(11), 1828–1835. 10.1093/scan/nsu020 24478326 PMC4221227

[dev22349-bib-0025] Groppe, S. E. , Gossen, A. , Rademacher, L. , Hahn, A. , Westphal, L. , Gründer, G. , & Spreckelmeyer, K. N. (2013). Oxytocin influences processing of socially relevant cues in the ventral tegmental area of the human brain. Biological Psychiatry, 74(3), 172–179. 10.1016/j.biopsych.2012.12.023 23419544

[dev22349-bib-0026] Haber, S. N. , & Knutson, B. (2010). The reward circuit: Linking primate anatomy and human imaging. Neuropsychopharmacology, 35(1), 4–26. 10.1038/npp.2009.129 19812543 PMC3055449

[dev22349-bib-0027] Hahn, A. C. , & Perrett, D. I. (2014). Neural and behavioral responses to attractiveness in adult and infant faces. Neuroscience & Biobehavioral Reviews, 46(P4), 591–603. 10.1016/J.NEUBIOREV.2014.08.015 25199981

[dev22349-bib-0028] Heim, C. , Newport, D. J. , Mletzko, T. , Miller, A. H. , & Nemeroff, C. B. (2008). The link between childhood trauma and depression: Insights from HPA axis studies in humans. Psychoneuroendocrinology, 33(6), 693–710. 10.1016/j.psyneuen.2008.03.008 18602762

[dev22349-bib-0029] Heim, C. , Young, L. J. , Newport, D. J. , Mletzko, T. , Miller, A. H. , & Nemeroff, C. B. (2009). Lower CSF oxytocin concentrations in women with a history of childhood abuse. Molecular Psychiatry, 14(10), 954–958. 10.1038/mp.2008.112 18957940

[dev22349-bib-0030] Hofer, M. K. , Buckels, E. E. , White, C. J. M. , Beall, A. T. , & Schaller, M. (2018). Individual differences in activation of the parental care motivational system: An empirical distinction between protection and nurturance. Social Psychological and Personality Science, 9(8), 907–916. 10.1177/1948550617728994

[dev22349-bib-0031] Hurlemann, R. , Patin, A. , Onur, O. A. , Cohen, M. X. , Baumgartner, T. , Metzler, S. , Dziobek, I. , Gallinat, J. , Wagner, M. , Maier, W. , & Kendrick, K. M. (2010). Oxytocin enhances amygdala‐dependent, socially reinforced learning and emotional empathy in humans. The Journal of Neuroscience: The Official Journal of the Society for Neuroscience, 30(14), 4999–5007. 10.1523/JNEUROSCI.5538-09.2010 20371820 PMC6632777

[dev22349-bib-0032] Koch, S. B. J. , Van Zuiden, M. , Nawijn, L. , Frijling, J. L. , Veltman, D. J. , & Olff, M. (2016). Intranasal oxytocin administration dampens amygdala reactivity towards emotional faces in male and female PTSD patients. Neuropsychopharmacology, 41(6), 1495–1504. 10.1038/npp.2015.299 26404844 PMC4832009

[dev22349-bib-0033] Lanius, R. A. , Vermetten, E. , & Pain, C. (2010). The impact of early life trauma on health and disease. Cambridge University Press. 10.1017/CBO9780511777042

[dev22349-bib-0034] Leppanen, J. , Ng, K. W. , Tchanturia, K. , & Treasure, J. (2017). Meta‐analysis of the effects of intranasal oxytocin on interpretation and expression of emotions. Neuroscience and Biobehavioral Reviews, 78, 125–144. 10.1016/j.neubiorev.2017.04.010 28467893

[dev22349-bib-0035] Lieberz, J. , Scheele, D. , Spengler, F. B. , Matheisen, T. , Schneider, L. , Stoffel‐Wagner, B. , Kinfe, T. M. , & Hurlemann, R. (2020). Kinetics of oxytocin effects on amygdala and striatal reactivity vary between women and men. Neuropsychopharmacology, 45, 1134–1140. 10.1038/s41386-019-0582-6 31785587 PMC7235226

[dev22349-bib-0036] Meinlschmidt, G. , & Heim, C. (2007). Sensitivity to intranasal oxytocin in adult men with early parental separation. Biological Psychiatry, 61(9), 1109–1111. 10.1016/j.biopsych.2006.09.007 17141739

[dev22349-bib-0037] Montoya, E. R. , & Bos, P. A. (2017). How oral contraceptives impact social‐emotional behavior and brain function. Trends in Cognitive Sciences, 21(2), 125–136. 10.1016/j.tics.2016.11.005 28089524

[dev22349-bib-0038] Montoya, E. R. , Bos, P. A. , Terburg, D. , Rosenberger, L. A. , & van Honk, J. (2014). Cortisol administration induces global down‐regulation of the brain's reward circuitry. Psychoneuroendocrinology, 47, 31–42. 10.1016/j.psyneuen.2014.04.022 25001954

[dev22349-bib-0039] Nawijn, L. , Krzyzewska, I. M. , van Zuiden, M. , Henneman, P. , Koch, S. B. J. , Mul, A. N. , Frijling, J. L. , Veltman, D. J. , Mannens, M. M. A. M. , & Olff, M. (2019). Oxytocin receptor gene methylation in male and female PTSD patients and trauma‐exposed controls. European Neuropsychopharmacology, 29(1), 147–155. 10.1016/j.euroneuro.2018.10.006 30415783

[dev22349-bib-0040] Olff, M. , Frijling, J. L. , Kubzansky, L. D. , Bradley, B. , Ellenbogen, M. A. , Cardoso, C. , Bartz, J. A. , Yee, J. R. , & van Zuiden, M. (2013). The role of oxytocin in social bonding, stress regulation and mental health: An update on the moderating effects of context and interindividual differences. Psychoneuroendocrinology, 38(9), 1883–1894. 10.1016/j.psyneuen.2013.06.019 23856187

[dev22349-bib-0041] Opacka‐Juffry, J. , & Mohiyeddini, C. (2012). Experience of stress in childhood negatively correlates with plasma oxytocin concentration in adult men. Stress, 15(1), 1–10. 10.3109/10253890.2011.560309 21682649

[dev22349-bib-0042] Peciña, S. , Smith, K. S. , & Berridge, K. C. (2006). Hedonic hot spots in the brain. The Neuroscientist: A Review Journal Bringing Neurobiology, Neurology and Psychiatry, 12(6), 500–511. 10.1177/1073858406293154 17079516

[dev22349-bib-0043] Reniers, R. L. E. P. , Völlm, B. A. , Elliott, R. , & Corcoran, R. (2014). Empathy, ToM, and self‐other differentiation: An fMRI study of internal states. Social Neuroscience, 9(1), 50–62. 10.1080/17470919.2013.861360 24294841

[dev22349-bib-0044] Riem, M. M. E. , Bakermans‐Kranenburg, M. J. , Pieper, S. , Tops, M. , Boksem, M. A. S. , Vermeiren, R. R. J. M. , Van Ijzendoorn, M. H. , & Rombouts, S. A. R. B. (2011). Oxytocin modulates amygdala, insula, and inferior frontal gyrus responses to infant crying: A randomized controlled trial. Biological Psychiatry, 70(3), 291–297. 10.1016/j.biopsych.2011.02.006 21470595

[dev22349-bib-0045] Riem, M. M. E. , van Ijzendoorn, M. H. , Tops, M. , Boksem, M. A. S. , Rombouts, S. A. R. B. , & Bakermans‐Kranenburg, M. J. (2012). No laughing matter: Intranasal oxytocin administration changes functional brain connectivity during exposure to infant laughter. Neuropsychopharmacology, 37(5), 1257–1266. 10.1038/npp.2011.313 22189289 PMC3306887

[dev22349-bib-0046] Riem, M. M. E. , Van IJzendoorn, M. H. , Tops, M. , Boksem, M. A. S. , Rombouts, S. A. R. B. , & Bakermans‐Kranenburg, M. J. (2013). Oxytocin effects on complex brain networks are moderated by experiences of maternal love withdrawal. European Neuropsychopharmacology, 23(10), 1288–1295. 10.1016/j.euroneuro.2013.01.011 23453164

[dev22349-bib-0047] Scheele, D. , Plota, J. , Stoffel‐Wagner, B. , Maier, W. , & Hurlemann, R. (2016). Hormonal contraceptives suppress oxytocin‐induced brain reward responses to the partner's face. Social Cognitive and Affective Neuroscience, 11(5), 767–774. 10.1093/scan/nsv157 26722017 PMC4847696

[dev22349-bib-0048] Seeley, W. W. , Menon, V. , Schatzberg, A. F. , Keller, J. , Glover, G. H. , Kenna, H. , Reiss, A. L. , & Greicius, M. D. (2007). Dissociable intrinsic connectivity networks for salience processing and executive control. Journal of Neuroscience, 27(9), 2349–2356. 10.1523/JNEUROSCI.5587-06.2007 17329432 PMC2680293

[dev22349-bib-0049] Shamay‐Tsoory, S. G. , & Abu‐Akel, A. (2016). The social salience hypothesis of oxytocin. Biological Psychiatry, 79(3), 194–202. 10.1016/j.biopsych.2015.07.020 26321019

[dev22349-bib-0050] Shamay‐Tsoory, S. G. , Abu‐Akel, A. , Palgi, S. , Sulieman, R. , Fischer‐Shofty, M. , Levkovitz, Y. , & Decety, J. (2013). Giving peace a chance: Oxytocin increases empathy to pain in the context of the Israeli‐Palestinian conflict. Psychoneuroendocrinology, 38(12), 3139–3144. 10.1016/j.psyneuen.2013.09.015 24099859

[dev22349-bib-0051] Spreckelmeyer, K. N. , Krach, S. , Kohls, G. , Rademacher, L. , Irmak, A. , Konrad, K. , Kircher, T. , & Gründer, G. (2009). Anticipation of monetary and social reward differently activates mesolimbic brain structures in men and women. Social Cognitive and Affective Neuroscience, 4(2), 158–165. 10.1093/scan/nsn051 19174537 PMC2686229

[dev22349-bib-0052] Strathearn, L. (2011). Maternal neglect: Oxytocin, dopamine and the neurobiology of attachment. Journal of Neuroendocrinology, 23(11), 1054–1065. 10.1111/j.1365-2826.2011.02228.x 21951160 PMC3319675

[dev22349-bib-0053] Thombs, B. D. , Bernstein, D. P. , Lobbestael, J. , & Arntz, A. (2009). A validation study of the Dutch Childhood Trauma Questionnaire‐Short Form: Factor structure, reliability, and known‐groups validity. Child Abuse and Neglect, 33(8), 518–523. 10.1016/j.chiabu.2009.03.001 19758699

[dev22349-bib-0054] Tzourio‐Mazoyer, N. , Landeau, B. , Papathanassiou, D. , Crivello, F. , Etard, O. , Delcroix, N. , Mazoyer, B. , & Joliot, M. (2002). Automated anatomical labeling of activations in SPM using a macroscopic anatomical parcellation of the MNI MRI single‐subject brain. NeuroImage, 15(1), 273–289. 10.1006/nimg.2001.0978 11771995

[dev22349-bib-0055] Watson, D. , Clark, L. A. , & Tellegen, A. (1988). Development and validation of brief measures of positive and negative affect: The PANAS scales. Journal of Personality and Social Psychology, 54(6), 1063–1070. 10.1037/0022-3514.54.6.1063 3397865

[dev22349-bib-0056] Witteman, J. , Van IJzendoorn, M. H. , Rilling, J. K. , Bos, P. A. , Schiller, N. O. , & Bakermans‐Kranenburg, M. J. (2019). Towards a neural model of infant cry perception. Neuroscience and Biobehavioral Reviews, 99, 23–32. 10.1016/j.neubiorev.2019.01.026 30710581

