# Supplementary material for: Preliminary data on oxytocin modulation of neural reactivity in women to emotional stimuli of children depending on childhood emotional neglect
Source: Dev Psychobiol. 2022 Nov 25;65(1):e22349. doi: 10.1002/dev.22349 (PMC9828591; doi:10.1002/dev.22349)
Supplement: Supplementary file 1 — Supplementary materials [file DEV-65-0-s001.docx]

**Supplementary materials**

*Accompanying Meier et al.* Preliminary data on oxytocin modulation of neural reactivity in women to emotional stimuli of children depending on childhood emotional neglect

**Methods**

*Post-hoc connectivity analysis*

A post-hoc connectivity analysis was conducted to investigate connectivity changes of the vmPFC and sPFCwith the amygdala and hippocampus under OXT vs. PLC and depending on childhood emotional neglect (CTQ-EN). A new mask was created of the vmPFC seed region using a 10mm sphere around the peak activation (x,y,z= -4 54 -10) based on the task effect (positive > neutral). All other masks remained the same. For both the OXT and PLC session, we performed a generalized form of context dependent psychophysiological interaction analysis (gPPI) (McLaren et al., 2012), using the gPPI toolbox (version 13.1, https://www.nitrc.org/projects/gppi). First, for each participant and each prefrontal seed region the first eigenvector (physiological factor) was extracted and adjusted for average task activation. Second, for each seed region and participant, a first-level gPPI model was created, modeling the physiological factor, the task conditions (psychological factor) and the interactions between the task conditions and physiological factor, to test for connectivity related to the interaction between the two factors. Subsequently, contrast images were created comparing connectivity during the neutral condition with baseline, and during the positive condition with baseline. These contrast images were submitted into second-level paired sample t-tests to investigate connectivity differences of the seed regions between the OXT and PLC session during the neutral and positive condition.

Due to the exploratory nature of the analysis, we focused exclusively on connectivity between the seed regions and the amygdala and hippocampus. Finally, average values of the connectivity analysis were extracted from the amygdala and hippocampus ROIs and correlated with CTQ-EN scores. At whole brain level, all results of the paired t-tests were assessed using a FWE correction. Small-volume corrections were applied at the level of the amygdala and hippocampus ROIs. To control for multiple comparison in the correlations with CTQ-EN the alpha level was set to α = 0.01.

**Results**

*

*

**Fig 4. Ratings of compassion for all stimuli types and by drug.** Participants rated the stimuli significantly different on compassion depending on valence. Drug had no effect on compassion ratings. *** = *p* < .0001.

*Analysis of inter-individual differences - PCAT*

To test whether caregiving motivation was a predictor of OXT effects on neural activity, PCAT-n and PCAT-p scores were added as a covariate in the separate repeated measures ANOVAs with the extracted values of ROIs that were found to be significant in the emotion specific t-tests (positive > neutral; negative > neutral), and the functional ROI extracted from the sPFC. In the validation study Cronbach’s α of the PCAT-n was 0.88 and 0.90 for the PCAT-p (Hofer et al., 2018). For the current sample we obtained a Cronbach’s α of 0.75 and 0.84 respectively. Mean score on the PCAT-n was m= 3.7 (sd., 0.76; range, 1.83 – 4.83) and m= 3.68 (sd., 0.84; range, 2.25 – 5) on the PCAT-p.

## PCAT-n

The PCAT-n significantly interacted with OXT administration in the putamen (F(1,20) = 4.99, *p* = .037 , η^2^ = .20), but not in any of the selected ROI’s (all *p* > .05). To test for the direction of this effect, the PCAT-n was added in a correlational analysis with the extracted values of the putamen ROI. A significant negative correlation was found between PCAT-n and activation of the putamen for neutral images after placebo (r = -.452, *p* = .035), but not after OXT (r = .276, *p* = .214). The correlations for positive (placebo: r = .-147, *p* = .515; OXT: r = .200, *p* = .373) and negative images (placebo: r = .-221, *p* = .323; OXT: r = .266, *p* = .231) were in the same direction, but not significant.

## PCAT-p

No significant interaction between the PCAT-p scale, OXT administration and neural activity was found in any of the ROIs.
